# e-Biologics: Fabrication of Sustainable Electronics with “Green” Biological Materials

**DOI:** 10.1128/mBio.00695-17

**Published:** 2017-06-27

**Authors:** Derek R. Lovley

**Affiliations:** Department of Microbiology, University of Massachusetts, Amherst, Massachusetts, USA; UT Southwestern Medical Center Dallas

**Keywords:** bioelectronics, biofilms, biomineralization, conductive proteins, electromicrobiology, electron transport, microbial nanowires

## Abstract

The growing ubiquity of electronic devices is increasingly consuming substantial energy and rare resources for materials fabrication, as well as creating expansive volumes of toxic waste. This is not sustainable. Electronic biological materials (e-biologics) that are produced with microbes, or designed with microbial components as the guide for synthesis, are a potential green solution. Some e-biologics can be fabricated from renewable feedstocks with relatively low energy inputs, often while avoiding the harsh chemicals used for synthesizing more traditional electronic materials. Several are completely free of toxic components, can be readily recycled, and offer unique features not found in traditional electronic materials in terms of size, performance, and opportunities for diverse functionalization. An appropriate investment in the concerted multidisciplinary collaborative research required to identify and characterize e-biologics and to engineer materials and devices based on e-biologics could be rewarded with a new “green age” of sustainable electronic materials and devices.

## “GE, WE BRING GOOD THINGS TO LIFE”—ADVERTISING SLOGAN

We know electronics can bring good things to life. Can life return the favor and bring good things to electronics? The expanding development of small electronic devices and the “Internet of things” are revolutionizing society, but not necessarily in a sustainable manner. The mining and processing of common inorganic electronic component materials, such as high-grade silicon and metals, are energy intensive and environmentally invasive. The production of synthetic organic conductive materials requires high energy inputs and/or harsh chemical processes. Most electronic devices are now considered “disposable,” but they contain toxic metals and organics, are difficult to recycle, and are not biodegradable. Therefore, it is not surprising that electronic waste (e-waste) is becoming a substantial environmental and health concern (1, 2).

What if electronic components and devices could be made from sustainable organic feedstocks or from carbon dioxide and renewable electricity? What if they could be synthesized with no toxic chemicals at room temperature with water as the “solvent”? What if the electronic materials produced this way were completely nontoxic, and when your electronic device was broken or outdated, these components could be thrown on your compost pile or converted to methane at the local municipal waste treatment plant?

This green electronic future may be possible with e-biologics (biologically produced electronic materials and related biomimetics). Microbes are one of the most promising catalysts for fabricating e-biologics, in part because of their highly evolved capacity to work at the nanoscale desirable for most electronic devices, as well as the ease of sustainably growing microbes on renewable feedstocks for inexpensive mass production. New classes of microbially produced minerals and proteins with not only conductive but also transistor and supercapacitor properties have been discovered in the last several years (Table 1). In addition to their superior green credentials, e-biologics may, in some instances, have advantages over traditional electronic components in size, physical properties, and electronic performance. Highlights of just some of the possibilities follow.

**TABLE 1:** e-Biologic types, fabrication methods, potential applications, and potential advantages over abiotic materials

Material	Fabrication method	Potential applications	Potential advantages over abiotic materials			
			Reduction in:			Flexibility in product design^c^
			Energy^a^	Harsh chemicals^b^	Toxic waste	
Metal/metalloid precipitates	Microbially mediated precipitation of soluble forms of metals/metalloids	Nanowires, transistors, capacitors	+	+		
Protein scaffolds for metals	*In vitro* assembly of peptides	Nanowires, capacitors	+	+		+
Lipid-cytochrome filaments	Outer membrane extensions dried and chemically fixed	Nanowires, transistors	+		+	
Electrically conductive pili	Microbial expression from native or synthetic PilA monomer gene	Nanowire electrical connections, conductive composite materials, nanosensors, transistors, capacitors	+	+	+	+
Self-assembling conductive protein wires	*In vitro* assembly of peptides	Nanowire electrical connections, conductive composite materials, nanosensors, transistors, capacitors	+	+	+	+
Living biofilms	Cell growth	Conductive “polymers” and circuits with potential for self-repair, sensors, biological computers	+	+	+	+

a Energy required for obtaining feedstock and fabrication of the material.

b Chemicals for synthesis.

c Potential for modifying structure with diverse aptamers.

## MICROBIAL DEPOSITION OF METALLIC AND SEMIMETALLIC ELECTRONIC MATERIALS

The extent to which e-biologics may help meet the goal of a sustainable future for electronic devices depends on the approach taken. Until recently, most research has focused on using microorganisms to produce nano-size metallic or semimetallic material that can serve either as a conductor, as the sensing component in electronic sensors, or for electrical energy storage. A diversity of microbes or enzymes extracted from microbial cells can produce nanoparticles from a wide range of metals, including gold, silver, palladium, and platinum (3, 4). Sizes and shapes depend on the microorganisms and environmental conditions employed. Semiconductor materials such as metal sulfides, metal oxides, and elemental selenium can also be microbially produced either intracellularly or extracellularly (3). Remarkable in this regard is the formation of arsenic-sulfide nanotubes by *Shewanella* species from the reduction of As(V) to As(III), concomitant with the reduction of thiosulfate to sulfide (5, 6). The nanotubes (20 to 100 nm by ca. 30 µm) form extracellular networks, that upon aging are not only electrically conductive but also exhibit photoluminescence, photoactive, and transistor-like properties (5, 7).

These inorganic e-biologics are not completely green, because they contain toxic metals or metalloids. However, the biosynthetic route has the advantage that it can be carried out in aqueous medium at standard temperature and pressure, reducing energy requirements and eliminating harsh chemical treatments associated with more traditional fabrication approaches. Furthermore, the production of these e-biologics is powered with renewable organic substrates, which generate the catalysts (microbes and their enzymes) and the electron donor, typically NAD(P)H, that serves as the reductant for metal, metalloid, and sulfur components.

Furthermore, not all metal precipitates are toxic. *Leptothrix ochracea* oxidizes ferrous iron naturally dissolved in groundwater to produce nontoxic ferric nanoparticles that show promise as anode materials for lithium ion batteries due to their high capacity for discharge/charge (8, 9). An additional benefit is that the ferrous iron oxidation provides energy to support cell growth.

## PROTEIN SCAFFOLDS FOR ELECTRONIC MATERIALS

Another promising strategy for organizing conductive metals at the nanoscale is to exploit biological molecules as scaffolds for templating metals or minerals. This approach has been intensively investigated with DNA, but DNA is fragile, and protein-based wires are expected to be more stable (10). Amyloid proteins can assemble *in vitro* into nanofilaments that are micrometers long and highly robust, withstanding high temperatures (ca. 100°C), pH extremes, common protein denaturants, and organic solvents (10). The filaments are typically poorly conductive, but simply introducing a cysteine into an amyloid monomer expressed in *Escherichia coli* yields a filament that binds gold and silver, creating wires (final diameter of 100 nm) with the conductivity and ohmic behavior of a metal wire (10). A diversity of other peptides that assemble into similar nanofilaments can also be decorated with metals to produce conductive nanowires that may have applications as energy storage devices (11).

The need for precious metals to confer conductivity to an amyloid protein nanowire was eliminated with a chimeric protein in which a portion of a microbial rubredoxin was fused with a portion of a fungal prion protein (12). Electrons hop over the ca. 1 nm separating the individual iron molecules of each monomer in the resultant nanowires (5 nm by 12 µm). The electron transport properties of individual wires were not reported, but nanowire networks were conductive, effectively transporting electrons between electrodes and a laccase enzyme (12).

## ORGANIZING ELECTRON TRANSPORT PROTEINS IN LIPID MEMBRANES

Nanowires that rely on electron hopping between iron-based electron transport proteins can also be produced from cytochrome-rich membrane extensions of *Shewanella oneidensis* (13–16). In live cells, the cytochromes are spaced too far apart to support long-range electron hopping along the length of the filaments (17). However, when the filaments are sheared from the cells, dried, and chemically fixed, they exhibit conductivities that are high (up to 1 S cm^−1^) for a biologically produced organic material (14). It is likely that the substantial shrinkage of the filaments associated with drying and fixation (from >500 nm in the hydrated physiological state down to 10 nm) brings the cytochromes close enough together (ca. 1 nm) to facilitate the proposed hopping mechanism of electron transport (13, 18, 19).

The conductive properties of the dried filaments, coupled with a mechanical strength comparable to those of organic polymers (20) and transistor-like response of individual filaments (15), suggest that they might be suitable substitutes for organic semiconducting nanomaterials in electronic devices such as biosensors, organic light-emitting diodes (LEDs), and organic solar cells (15, 20). Strategies for mass production of the wires and their alignment in devices are available (15). The potential for making *S. oneidensis* wires without fixation with toxic glutaraldehyde should be examined, as eliminating this step from the process would increase the attractiveness of these filaments as a green electronic material.

## MICROBIALLY PRODUCED CONDUCTIVE PROTEIN NANOWIRES

Electrically conductive pili (e-pili) are another type of “microbial nanowire,” but they are completely different in form and function than those that can be produced with *S. oneidensis* (21). Whereas *S. oneidensis* wires are comprised of a mixture of lipids and diverse proteins, purified e-pili are comprised of a single peptide monomer, PilA, which is homologous to the pilin monomer of many type IV pili (22, 23). A diversity of microorganisms appear to be capable of expressing e-pili (24–26), but to date, only the e-pili of *Geobacter sulfurreducens* have been studied in detail. The thin (ca. 3-nm) *G. sulfurreducens* e-pili can transport electrons along their length of up to 10 to 30 µm (27–29). Networks of e-pili transport electrons over centimeters (30). Such long-range electron transport in a biological protein is unprecedented and “surprising” (31). Typically, electron transport of ca. 10 nm is considered to be a great distance for protein complexes (31, 32).

The mechanisms for long-range electron transport for e-pili are still a matter of debate (21, 33). However, there is consensus among those that have studied highly purified individual e-pili that, as was originally proposed (22, 30), electrons are transported along the e-pilus protein filament themselves, not metals or redox-active proteins associated with the e-pili (27, 29). It is also clear that it is possible to change the properties of e-pili with simple genetic manipulation of the PilA sequence. For example, by manipulating the density of aromatic rings in PilA (28, 34), it is possible to tune the conductivity of *G. sulfurreducens* over a broad range (1 mS/cm to 1 kS/cm). Conductivity can also be tuned over 3 to 4 orders of magnitude simply by changing the pH at which e-pili are prepared (27, 28, 30).

e-Pili are highly durable, yet biodegradable, and contain no toxic components, eliminating e-waste. One advantage of e-pili over many nonbiological electronic materials is that they can be processed and function well in water and they are stable over a wide range of pH values (from pH 2 to 10). However, if needed, e-pili can also be employed in processing approaches common with more traditional electronic materials because they are stable when dried under vacuum and in a diversity of organic solvents (Y.-L. Sun, University of Massachusetts, personal communication). Furthermore, e-pili have the potential to be functionalized by the addition of amino acid sequences that can function as linkers to enhance attachment to substrates, serve as aptamers for sensing applications, and can covalently link e-pili to polymeric materials to produce conductive composites. e-Pili can readily be mass produced with high uniformity from inexpensive, renewable feedstocks, such as acetate, with energy inputs for fabrication estimated to be 100-fold less than that required for processing traditional electronic materials (V. Zhirnov, Semiconductor Research Corporation, personal communication).

## SELF-ASSEMBLING CONDUCTIVE PROTEIN NANOWIRES

The production of electrically conductive protein nanowires could be simplified if the nanowires would correctly assemble *in vitro* from peptide monomers. Rational design of such structures will require a better understanding of the e-pilus structure and mechanisms for electron transfer. However, progress is already being made. Peptides derived from type IV pili can assemble into nanotubes that differ in structure from the type IV pili but maintain a similar diameter and some external physical/chemical characteristics (35). Peptide sequences of 20 amino acids designed from portions of the *G. sulfurreducens* PilA sequence yielded fibers, but conductive properties still need to be evaluated (36). The short peptide glycine-phenylalanine-proline-arginine-phenylalanine-alanine-glycine-phenylanine-proline self-assembled into helical filaments of single monomer diameters that promoted π-π stacking of the phenylalanines in a manner similar to that proposed (30, 37) to confer conductivity to *G. sulfurreducens* e-pili (38). Films of the fibers were conductive (38). Various other short peptides will, under the appropriate conditions, assemble into networks of nanofilaments that exhibit conductivity, which is enhanced by increasing the π-π stacking of aromatic amino acids (39–41). These results further suggest that it may eventually be possible to design self-assembling peptides that yield protein nanowires with conductivities comparable to those of microbially assembled e-pili.

## CELLS AND BIOFILMS AS LIVING ELECTRONIC DEVICES

An additional step toward green electronics is to move beyond mere incorporation of e-biologics as components of electronic devices and grow a living device that is electronically responsive. *G. sulfurreducens* biofilms are as naturally conductive as some synthetic organic conducting polymers and exhibit transistor-like as well as supercapacitor properties (30, 42, 43). *E. coli* biofilms can be made conductive by introducing metal-binding motifs to curli fibers and exposing the biofilms to gold nanoparticles (44). Peptides that facilitate attachment of semiconducting materials or promote mineral nucleation on curli fibers are also available (44, 45). Multiple strategies have been developed for controlling the spacing of the functionalized regions along the length of the curli fibers, further expanding the range of filament performance (44). Living biofilms can potentially be programmed to extend, and in the case of e-pili also retract, filament networks in response to chemical, light, or electrical signals. Encasing cells in polymeric materials can help stabilize electronic biofilms (46), and genetic modification greatly increased the cohesiveness and conductivity of the electronic biofilm material (47). Three-dimensional (3-D) printing of cells can facilitate high-resolution patterning (48). When cell viability is maintained, biofilm-based electronic devices have the capacity for self-repair. The ability of some microorganisms to generate a current makes self-powering devices possible (49). Living sensors and microbial computers can be constructed by introducing genetic circuits that control when the cells can produce an electrical signal (50, 51).

## CONCLUSIONS AND OUTLOOK

It remains to be seen whether commercially viable applications will emerge from e-biologics. The available research shows that the potential societal benefits on both the front end (fabrication) and back end (disposal) could be enormous in terms of resources saved and reduction of toxic e-waste. However, the sustainability of e-biologics is unlikely to be the driver for their initial incorporation in electronic devices. More likely, early adoption will require enhanced performance over existing materials. For example, conductive proteins may be superior for some sensing applications because it should be easier to functionalize protein nanowires with diverse aptamers that interact with analytes than comparable nonbiological nanowire materials, such as carbon nanotubes or silicon nanowires. Additional benefits of e-biologics may be realized in the development of sensors for biomedical and environmental monitoring where there is a premium on biocompatibility and/or the ability to function in water. e-Pili are stable and function well in water, whereas silicon nanowires dissolve (52). Billions of years of evolution have optimized some e-biologics for electron-based electrical communication with cellular components, a key advantage for some biomedical applications.

The search for e-biologics in nature has been very limited and should be expanded to identify unique materials that may expand applications. Intriguing in this regard are the cable bacteria, which form multicellular chains that appear to be capable of transporting electrons over centimeters (53). Their mechanism for electron transport is not known, but it might involve some novel form of conductive biological material. Simple screening tools for prospecting for new electronic materials within the diverse world of difficult-to-culture microorganisms (25) are helpful, but ultimately, sophisticated characterization techniques, not commonly accessible to microbiologists, are essential to evaluate these materials. Interdisciplinary collaboration between microbiologists, physicists, material scientists, biochemists, and electrical engineers will be necessary to move this field forward and make the applications a reality.
